# Using machine learning to link the influence of transferred *Agrobacterium rhizogenes* genes to the hormone profile and morphological traits in *Centella asiatica* hairy roots

**DOI:** 10.3389/fpls.2022.1001023

**Published:** 2022-09-02

**Authors:** Miguel Angel Alcalde, Maren Müller, Sergi Munné-Bosch, Mariana Landín, Pedro Pablo Gallego, Mercedes Bonfill, Javier Palazon, Diego Hidalgo-Martinez

**Affiliations:** ^1^Department of Biology, Healthcare and the Environment, Faculty of Pharmacy and Food Sciences, University of Barcelona, Barcelona, Spain; ^2^Department of Evolutionary Biology, Ecology and Environmental Sciences, Faculty of Biology, University of Barcelona, Barcelona, Spain; ^3^Department of Pharmacology, Pharmacy and Pharmaceutical Technology, Group I+D Farma (GI-1645), Faculty of Pharmacy, University of Santiago, Santiago de Compostela, Spain; ^4^Agrobiotech for Health, Department of Plant Biology and Soil Science, Faculty of Biology, University of Vigo, Vigo, Spain; ^5^Department of Plant and Microbial Biology, University of California, Berkeley, Berkeley, CA, United States

**Keywords:** *Agrobacterium rhizogenes*, plant hormones, hairy root cultures, *Centella asiatica*, centellosides, machine learning, random forest

## Abstract

Hairy roots are made after the integration of a small set of genes from *Agrobacterium rhizogenes* in the plant genome. Little is known about how this small set is linked to their hormone profile, which determines development, morphology, and levels of secondary metabolite production. We used *C. asiatica* hairy root line cultures to determine the putative links between the *rol* and *aux* gene expressions with morphological traits, a hormone profile, and centelloside production. The results obtained after 14 and 28 days of culture were processed *via* multivariate analysis and machine-learning processes such as random forest, supported vector machines, linear discriminant analysis, and neural networks. This allowed us to obtain models capable of discriminating highly productive root lines from their levels of genetic expression (*rol* and *aux* genes) or from their hormone profile. In total, 12 hormones were evaluated, resulting in 10 being satisfactorily detected. Within this set of hormones, abscisic acid (ABA) and cytokinin isopentenyl adenosine (IPA) were found to be critical in defining the morphological traits and centelloside content. The results showed that IPA brings more benefits to the biotechnological platform. Additionally, we determined the degree of influence of each of the evaluated genes on the individual hormone profile, finding that *aux1* has a significant influence on the IPA profile, while the *rol* genes are closely linked to the ABA profile. Finally, we effectively verified the gene influence on these two specific hormones through feeding experiments that aimed to reverse the effect on root morphology and centelloside content.

## Introduction

Plant specialized metabolism is the source of a plethora of bioactive compounds, some of which are uncommon and with important pharmacological activities (Rai et al., 2017). The overuse of medicinal plants and the fact that some of their bioactive compounds are only found in trace amounts in plant tissues has prompted the search for alternative sources of these compounds (Chen et al., 2016).

This is the case with *Centella asiatica* (L.) Urban, which has been used in traditional medicine to treat several chronic diseases since ancient times (Prasad et al., 2019). *C. asiatica* extracts have antidepressant, antiepileptic, antidiabetic, anxiolytic, neuroprotective, antioxidant, antiulcer, antitumor, anti-inflammatory, and healing properties (Gallego et al., 2014; Sun et al., 2020; Arribas-López et al., 2022), about 139 metabolites has been isolated from this plant most of them extracted from leaves and roots (Kunjumon et al., 2022). *C. asiatica* is well known for the accumulation of triterpenoid centelloside, such as madecasosside and asiaticoside, as well as its relevant aglycones madecassic and asiatic acid (Joshi and Chaturvedi, 2013), which contribute to its clinical efficacy (Prakash et al., 2017). As a result, host plants have been over-exploited, and excessive uprooting has put *C. asiatica* in danger of extinction (Mangas et al., 2008). The chemical synthesis of centellosides is either impossible or economically unviable. Research has focused on the potential offered by plant cell culture technology, also known as Plant Biofactories, for efficient specialized metabolite production, such as centellosides (Dhillon et al., 2017).

One of these technologies is the generation of hairy roots, which has been used in several biotechnological approaches for phytochemical production (Donini and Marusic, 2018; Roy, 2020). Hairy root cultures are induced in most dicotyledonous plants by incorporating a segment of *Agrobacterium rhizogenes* DNA (T-DNA) from the plasmid Ri-DNA into the plant cell genome, where the expression of the genes carried out by the T-DNA promotes rooting at the site of infection. Hairy roots can grow rapidly even in the absence of exogenous growth regulators, which is why they are widely used as a transgenic tool to produce specialized metabolites, therapeutic proteins, etc. (Gutierrez-Valdes et al., 2020).

The plasmid Ri-DNA of *A. rhizogenes* strain A4 contains two regions: TL-DNA and TR-DNA. The first contains four *rol* genes (rooting locus): A, B, C, and D, which improve plant cell susceptibility to auxins and cytokinins and are responsible for the formation of these roots (Supplementary Figure 1; Mauro et al., 2017). However, little is known about the molecular changes induced in plant cells by the expression of *rol* genes into the plant genome. *rol*A is found on all Ri plasmids and encodes a small protein with a basic isoelectric point whose expression showed a dramatic reduction in several classes of hormones (Ozyigit et al., 2013). *rol*B may play a critical role in the early stages of hairy-root induction and is the most powerful inducer of secondary metabolism (Dilshad et al., 2021). *rol*C is considered the most conserved of all *rol* genes and has a minor impact on root formation (Makhzoum et al., 2013). T-DNA also contains genes that encode enzymes that direct the production of opines, which are synthesized and excreted by transformed cells and consumed as a source of carbon and nitrogen by *A. rhizogenes* (Matveeva and Otten, 2021). The second region of T-DNA is TR-DNA which contains genes related to auxin biosynthesis, known as *aux*1 and *aux*2. Both regions can be transferred to the nuclear genome of infected plant cells independently (Nemoto et al., 2009).

Most studies on the expression of *rol* and *aux* genes in hairy root cultures have focused on demonstrating their effect on specialized metabolism for increasing the production of phytochemicals, such as centelloside production in *C. asiatica* hairy root cultures (Dilshad et al., 2021), anthraquinones in *rol*A-transgenic tissues of *Rubia cordifolia* calli (Nemoto et al., 2009), or nicotine in tobacco hairy roots (Palazón et al., 1997). The *rol*B transformation was shown to induce resveratrol production in *Vitis amurensis* cells (Kiselev et al., 2007), and *rol*C gene expression was shown to be highly efficient for increasing the production of morphinan, tropane alkaloids (Cardillo et al., 2013; Hashemi and Naghavi, 2016), anthraquinones (Bulgakov et al., 2002).

On the contrary, the relationship between *rol* and *aux* genes expression and the hormonal profile, which is a determinant in root development and morphology (Wahby et al., 2012), has received little attention. As a result, we focus on analyzing the relationship between: (1) expression levels of different *rol* and *aux* genes, (2) morphological traits, (3) production of triterpene saponins and (4) hormonal profiling of various hairy root lines of *C. asiatica*. The combined analysis of these variables enabled us to generate machine learning models that allow for the discrimination of producing lines or lines with improved traits, either by the level of gene expression or hormonal profile. The extent to which *A. rhizogenes* genes influenced each of the hormones measured has been determined.

## Materials and methods

### Establishment of hairy root culture

The *A. rhizogenes* A4 strain was used in transformation experiments. Bacteria were grown for 48 h (OD 600 = 0.5–0.6) in liquid YEB (Yeast Extract Beef) medium at 28°C on a rotary shaker at 130 rpm. Explants for co-cultivation and hairy root induction were leaf segments from a *in-vitro* 2-month-old seedling of *C. asiatica*. The explants were cut into 1.5–2 cm^2^ disks with the tip of a scalpel containing a colony of *A. rhizogenes*, cultured at 25°C. All excised explants were then co-cultured in solid MS hormone-free media enriched with 3% sucrose and pH = 5.8. The explants were transferred to a fresh solid MS medium containing 500 mg/l cefotaxime after 48 h of cocultivation in the dark at 28°C. The emerging hairy roots were excised and transferred to a fresh solid MS medium containing 500 mg/l cefotaxime, where they were grown in darkness at 25°C on a rotary shaker. To eliminate the bacteria, this step was repeated every 2 weeks for about 2 months.

### Semiquantitative RT-PCR detection and expression of transgene integrations

Semiquantitative RT-PCR was used to detect the integration and expression of *A. rhizogenes* T-DNA genes (*rolA*, *rolB, rolC*, and *aux1*) at the transcript level in the studied transgenic clones, this analysis was previously perform in *C. asiatica* by Mangas et al. (2008). *PureLink RNA Mini Kit (Invitrogen)* was used to isolate RNA from 200 mg of fresh hairy roots lines according to the manufacturer’s instructions. The amount and quality of each RNA sample were determined using the *NanoDrop 2000 Spectrophotometer (Thermo Scientific)*. The integrity of the RNA was assessed using agarose gel electrophoresis. The total RNA at a fixed concentration (1.5 μg of RNA) was used as the template for the DNAse treatment. For this purpose, the required sample volume was calculated, taking into account the volume of DNAse I and buffer needed, and brought up to a final volume of 10 μl per sample with sterile H_2_O. After adding the DNAse mix, the samples were heated at 37°C for 30 min. Then, for each sample, 1 μl of 50 mM EDTA was added and incubated at 65°C for 10 min to inactivate it. First-strand cDNA was synthesized using the *SuperScriptTM IV First-Strand Synthesis System (Invitrogen)* kit and 2 μl of RNA according to the manufacturer’s instructions. Primer3Plus software^1^ was used to design PCR primers with G/C content and the presence of introns (Supplementary Table 1). A volume of 1 μl of cDNA products were amplified with 12.5 μl of Green Taq polymerase, 1 μl of each specific primer, and 9.5 μl of H_2_0 milliQ. A 5-min cycle at 94°C was followed by 60 s at 94°C, 30 s at 60°C, and 1 min at 72°C for 35 cycles, and then another 5-min cycle at 72°C. A no-sample negative control was always included in each set of reactions. PCR products were loaded onto 1% agarose gels in TAE buffer (1X), and pictures were taken using a Gel Logic 100 camera (KODAK). The bands were quantified using the Kodak Gel Logic 100 Digital Imaging System software (KODAK).

### Evaluation of some morphology parameters of hairy root lines

An inoculum of 10 mg fresh weight (FW) from each hairy root line was placed in plates with MS medium solid, and the cultures were maintained for two subcultures every 2 weeks at 25°C in dark conditions, as we had done in previous studies (Alcalde et al., 2022), before evaluating the growth parameters considered using three replicas of each line. The branching rate was defined as the number of lateral roots per cm of initial stem root (number of lateral roots/cm); the growth rate as the average length of the lateral roots (mm/day); and the biomass productivity as the final FW minus initial FW divided by the number of growing days (mg/day).

### Extraction and quantification of centellosides

Centellocide production was determined according to Hidalgo et al. (2016) and Alcalde et al. (2022) with slight modification. We weighted 0.5 g of freeze dry material (DW) of hairy roots and added 10 ml of methanol: H_2_O (9:1) suspension, which was sonicated for 1 h at room temperature. The following step was to centrifuge at 20,000 rpm for 10 min. After separating the supernatant, the previous step was repeated. The supernatants of the various samples were placed on porcelain mortars and evaporated at 38°C for approximately 24 h before being redissolved in 1.5 ml of methanol. The methanolic extract was filtered through a 0.22 μm filter for HPLC quantification of centellosides. The HPLC system consisted of a Waters 600 Controller pump, a Waters 717 Autosampler automatic injector, a Jasco variable length (UV) 1570 detector, and Borwin data analysis software version 1.5. At room temperature, a Lichrospher 100 RP18 5 μm column (250 × 0.4 mm) was used for gradient chromatography, as described in Supplementary Table 2. The mobile phase consisted of acetonitrile and ammonium phosphate (10 mM), which had been acidified with ortho-phosphoric acid to a pH of 2.5. The acidification improved the definition of the compound peaks. The flow rate was 1 ml/min, and the injection volume was 10 μl. The detector wavelength was set to 214 nm, 1.00 au/v, and the run time was 45 min. To quantify the centellosides (asiatic acid, madecassic acid, asiaticoside, and madecasoside), standards of these 4 compounds were used to prepare calibration curves at concentrations of 10, 25, 50, 100, 250, and 500 ppm.

### Hormonal profiling of hairy root lines

Samples were collected for hormonal profiling and immediately frozen in liquid nitrogen before being stored at −80°C for subsequent analyses. The endogenous concentrations of the compounds: abscisic acid (ABA), salicylic acid (SA), jasmonates (12-*oxo*-phytodienoic acid [OPDA], jasmonic acid [JA], and jasmonoyl isoleucine [JA-Ile]), cytokinins (2-isopentenenyladenine [2iP], IPA, *trans*-zeatin [*t*-Z], and *trans*-zeatin riboside [*t*-ZR]), the auxin indole 3-acetic acid (IAA), and gibberellins (GA_1_, GA_4,_ and GA_7_) were quantified using a protocol modified by Müller and Munné-Bosch (2011). For each hairy root line, 100 ± 5 mg samples were placed in liquid nitrogen in a 2 ml Eppendorf tube using the mixer mill MM400 (Retsch GmbH, Haan, Germany), and then extracted twice with extraction solvent (methanol:isopropanol:acetic acid in a proportion 50:49:1 [v/v/v] with 1% of glacial acetic acid) using ultrasonication (4–7°C). Deuterium-labeled compounds (Olchemim, Olomuc, Czech Republic) were used as internal standards for all phytohormones to estimate recovery rates for each sample. The quantifications were performed by preparing a calibration curve with each of the 13 compounds analyzed and calculating the compound/standard ratio using Analyst™ software (Applied Biosystems, Inc., Foster City, CA, United States). The results were expressed using the FW of the samples.

### Statistical analysis and machine learning models

The statistical analysis was carried out using GraphPad Prism version 6.04 for Windows, GraphPad Software, La Jolla California, United States.^2^ Data are presented as the mean ± standard deviation. For statistical comparison, a multifactorial ANOVA analysis was performed, followed by Tukey’s multiple comparison test. For morphologic traits, phytohormone concentration, and centelloside production, a p-value ≤ 0.05 was assumed to indicate a significant difference.

Machine learning models, principal component analysis (PCA), and Pearson correlation were performed using R Statistical Software (R Core Team, 2021). The caret package (Kuhn, 2021) was used to create LDA, SVM, RF, and ANN models, whereas randomForestSRC package (Ishwaran and Kogalur, 2022) was used to execute multivariate multiple regression models. A general scheme of the modeling process is presented in Supplementary Figure 9. For model validation, data split and repeated cross-validation methods were used, with 10-fold cross-validation repeated 5 times. Accuracy or coefficient of determination (R-squared) was used as metrics to evaluate the performance of each model, along with specificity and sensitivity. Factoextra (Kassambara and Mundt, 2020) package was used for the PCA, while corrplot package (Wei and Simko, 2021) was used for the calculation of the Pearson correlation coefficient (r). The multivariate multiple regression model’s decreased accuracy values were used to perform a hierarchical clustering analysis and were displayed as a heatmap. Datasets for model development and PCA analysis were preprocess by autoscaling method.

### Feeding experiment

To validate the model predictions and multivariate analysis results from the previous sections, we designed an experiment that consisted of supplying for 14 and 28 days ABA (13 and 1,300 ng/l) to the line L1 and IPA (1.5 and 150 ng/l) to the line L3. We used a single root to maximize visualization of the effect on growth and branching rates. ABA was bought to Sigma-Aldrich (Steinheim am Albuch, Germany) and IPA was bought to Cayman chemical (Ann Arbor, Michigan, USA). The stock solutions where prepared at 1 mg/ml in methanol followed by serial dilution prior to be added to culture media.

## Results

### Hairy root traits and centelloside content

**Figure 1:**
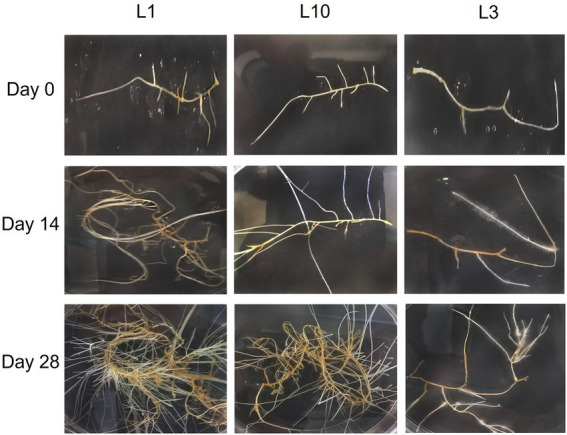
Average morphology of the different transformed hairy roots of *C. asiatica* at different stages of development.

**Figure 2:**
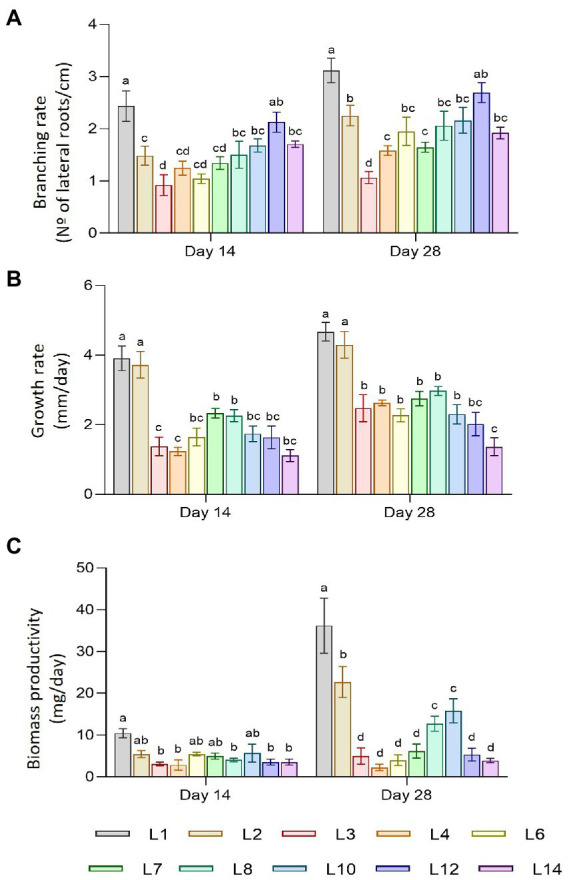
Morphological traits of hairy root lines of *C. asiatica*. **(A)** Branching rate. **(B)** Growth rate. **(C)** Biomass productivity. Data represent the mean ± SD of three replicates. Different letters show significant differences between hairy root lines. ns = no differences (*α* = 0.05).

The plant material used in this study consisted of 10 hairy root lines free of *Agrobacterium rhizogenes* (L1, L2, L3, L4, L6, L7, L8, L10, L12, and L14), which were cultivated for 2 and 4 weeks, before morphological traits and biomass production were registered, as described in the Material and methods section. Figure 1 shows the development of the root lines throughout the experiment. Most of the lines had the typical morphology of hairy roots, but there were noticeable differences in branching rate, growth rate, and biomass accumulation. Statistical differences were found between lines in terms of branching and growth rate, but there were no significant changes between sampling times. Figure 2A shows the branching rate, with L1 standing out from the others, followed by L12. L3 had the lowest branching values at 2 and 4 weeks of growth. In terms of growth rate (Figure 2B), two lines predominated: L1 and L2. Line L14 has the lowest growth rate value at the end of 4 weeks. Finally, we observed significant differences in biomass productivity (Figure 2C) between weeks 2 and 4 for lines L1, L2, L8, L10, and L12, with increases ranging from 2 to 5 times.

**Figure 3:**
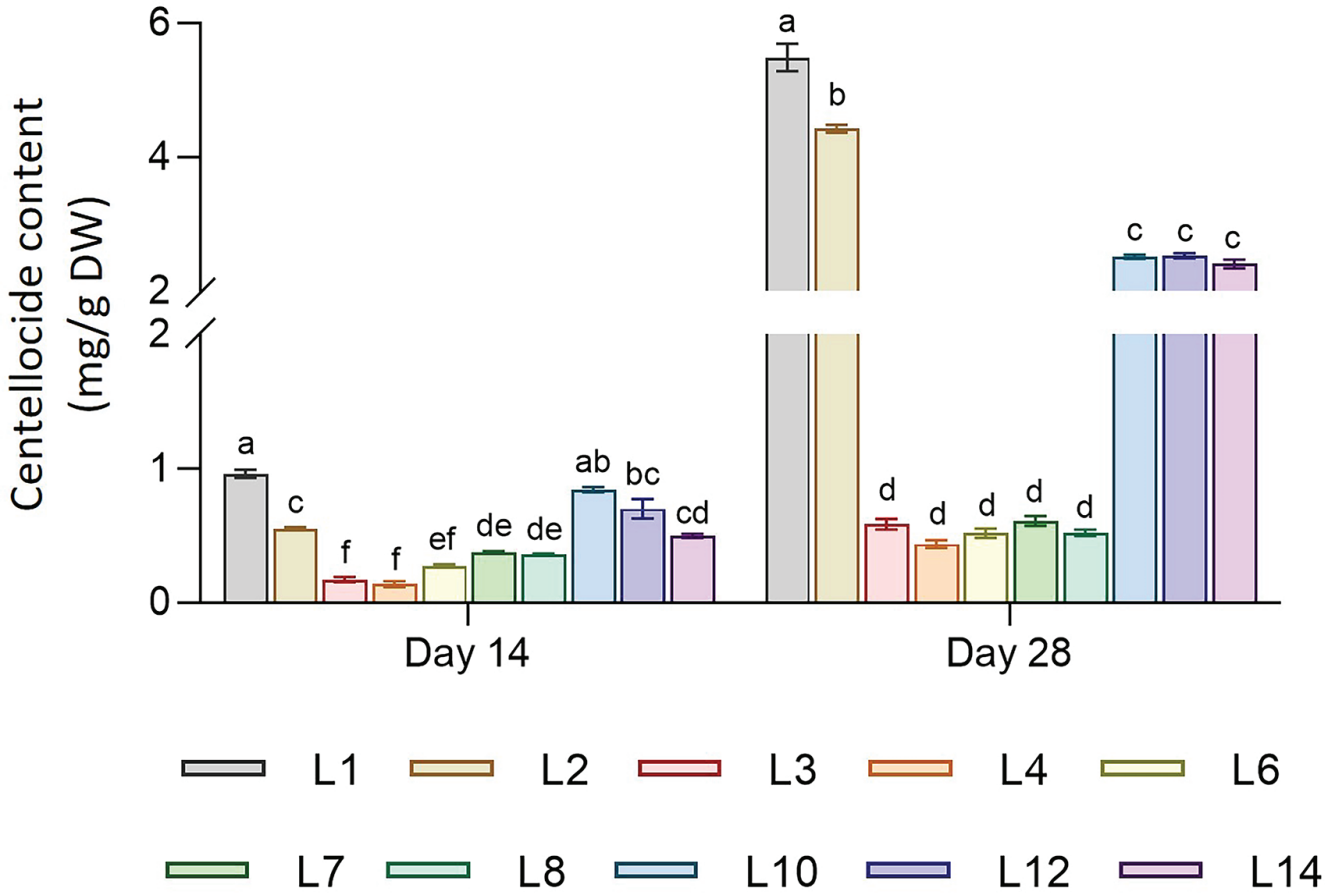
Centelloside content in mg/g DW of hairy root lines of *C. asiatica* measured as the sum of asiaticoside + madecassoside + asiatic acid + madecassic acid. Data represent the mean ± SD of three replicates. Different letters show significant differences between hairy root lines. ns = no differences (*α* = 0.05).

The total productivity of centelloside was expressed in two ways: mg per g of dry weight (DW) and mg per liter of culture medium (Figure 3). These results showed a similar profile between hairy root lines as the traits mentioned previously, as well as significant differences between sampling times. Lines 1 and 2 had the highest production values, followed by lines 10, 12, and 14. Lines 3 to 8 had the lowest values. The productivity range of centelloside values in mg/g DW oscillated between 0.14 ± 0.02 and 0.96 ± 0.03 after 14 days, and between 0.44 ± 0.27 and 5.49 ± 0.20 after 28 days. These results revealed the root lines’ various capacities to accumulate this type of compound over time. Some of them, such as L2, showed a content that was 8 times higher at 28 days than it was at 14 days, whereas line L7’s increase was only 0.7 times. In terms of centellosides profile, made cassoside was the main compound in almost all lines (Supplementary Figure 2). We can see from these results that the transformed root lines can be divided into at least 3 groups: L1 and L2, then L3, L4, L6, L7, and L8, and finally L10, L12, and L14.

### Gene expression

**Figure 4:**
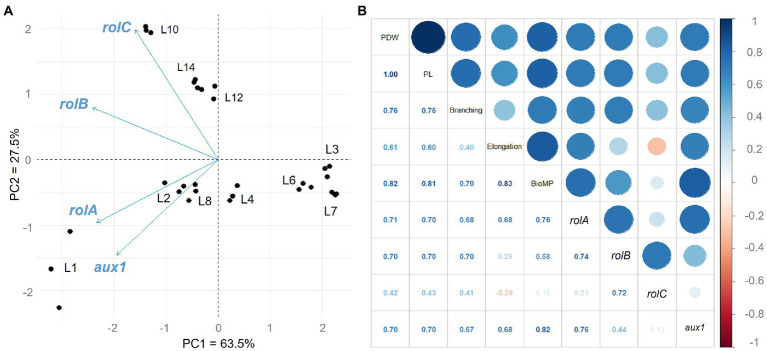
Gene expression analysis and correlation with morphological traits and centelloside production of hairy roots at 28 days of culture. **(A)** Biplot of Principal Component Analysis of the genes studied. **(B)** Pearson’s correlation analysis of gene expression, morphological traits, and centelloside production. PWD = centelloside production in mg/g, PL = centelloside production in mg/L, Elongation = growth rate, and BioMP = biomass productivity.

PCR (data not shown) and semi-quantitative RT-PCR were used to confirm the integration and expression of the *rol* and *aux* genes. Supplementary Figure 3 shows the results of semi-quantitative RT-PCR in 10 *C. asiatica* hairy root lines grown in MS basal medium after 28 days, with the 5.8 s rRNA used as a housekeeping gene for normalization. A principal component analysis (PCA) was used to investigate how the expression of these genes was related to hairy root traits and centelloside content. Figure 4A summarizes the information about hairy root samples and their multiple gene expression by two components: PC1 = 63.5% and PC2 = 27.5%, which account for 91% of the model’s total variance. L3, L4, L6, and L7 are the lines with the lowest expression of all genes, according to PC1. According to PC2, other subgroups can be seen within the lines with higher expression. The first (L10, L12, and L14) was associated with high expressions of the *rolC* and *rolB* genes, while the second (L1, L2, and L8) showed higher expressions of the *rolA* and *aux1* genes. A positive correlation was observed between centelloside content, branching, biomass productivity, *rol,* and *aux*1 genes (Figure 4B). The highest centelloside productions were strongly related to *rol*A (*r* = 0.71) and *aux*1 (*r* = 0.70) genes. The *rol*C was the least effective, with a slightly negative effect on elongation rates (*r* = −0.29). Similar behavior was observed for samples at 14 days (Supplementary Figure 4).

### Prediction of production degree based on gene expression

The statistically significant correlations and differences established the concept of identifying production lines based on their gene expression profile. We assigned the following tags to the subgroups that represent the centelloside content: HIGH (L1 and L2), MID (L10, L12, and L14), and LOW (L3, L4, L6, L7, and L8). Four different classification machine learning algorithms were tested on a dataset (Supplementary Table 3) containing gene expression information from 10 lines cultivated for 14 and 28 days, and accuracy was used to track the models’ performance (Table 1). The most accurate model was random forest (RF) with 91.33% correct classification, and the least accurate was artificial neural network (ANN) with 89.5% correct classification. The sensitivity range was 62.5 to 100%, with RF having the highest values, followed by linear discriminant analysis (LDA) and ANN, and supported vector machine (SVM) having the lowest values. Model specificity ranged between 78.6 and 100%. The importance of each gene for correct classification was calculated and ranked by the mean decrease in Gini. The *rol*C is at the top of the list, followed by *rol*A, *rolB*, and finally *aux*1 (Supplementary Figure 5A).

**Table 1:** Prediction of production degree based on gene expression and hormone profiles.

Model	Accuracy %	Sensitivity %			Specificity %		
		HIGH	MID	LOW	HIGH	MID	LOW
**Prediction of production degree based on gene expression**							
LDA	90.77	66.67	100	100	100	100	83.33
SVM	90.05	83.33	100	62.5	78.57	100	91.67
RF	91.33	83.33	100	87.5	92.9	100	91.7
ANN	89.5	66.67	100	100	100	100	83.33
**Prediction of production degree based on hormone profiles**							
LDA	82.57	100	100	50	75	100	100
SVM	100	100	100	100	100	100	100
RF	100	100	100	100	100	100	100
ANN	93.75	100	100	100	100	100	100

### Hormone profiles

The hormone profile of the hairy root lines was evaluated at 14 and 28 days, with 13 compounds measured, including ABA, SA, OPDA, JA, JA-Ile, 2iP, IPA, *t*-Z, *t*-ZR, IAA, GA_1_, GA_4_, and GA_7_. GA_4_ was detected, but GA_1_ and GA_7_ values were below the detection threshold for all rhizoclones (Supplementary Figure 6). LOW centelloside producer lines had higher concentrations of ABA, SA, JA-Ile, and IAA, especially at 4 weeks of growth. MID centelloside producer lines had the highest concentration of 2-iP throughout the experiment. HIGH producer centelloside lines had the highest concentration value for IPA. The other hormones (JA, OPDA, *t*-Z, *t*-ZR, and GA_4_) showed different values depending on the week of growth for each transformed root line.

**Figure 5:**
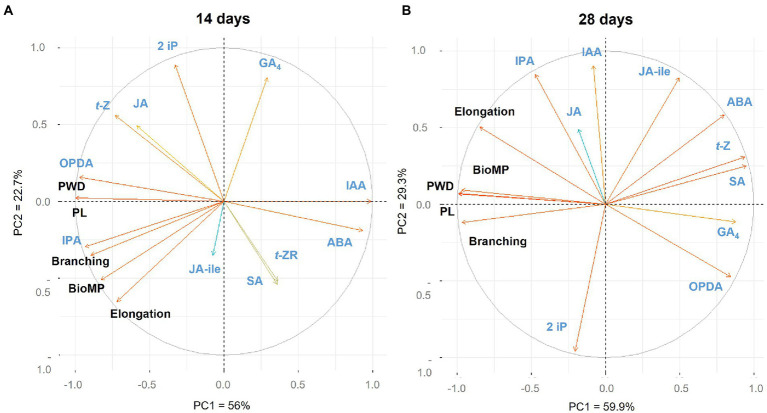
Loading plot of hormonal profiling, morphological traits and centelloside production of hairy roots cultures **(A)** at 14 days and **(B)** at 28 days. PWD = centelloside production in mg/g, PL = centelloside production in mg/L, Elongation = growth rate, and BioMP = biomass productivity.

The loadings plot in Figure 5 depicts the relationship between hairy root traits, centelloside contents, and hormone profile at various sampling times. The PCA was composed by nine components, where PC1 covered the 56% and PC2 = 22.7% which account for 78.9% of the model’s total variance. At 14 days (Figure 5A; Supplementary Figure 7), the only hormone with a strong positive correlation (*r* > 0.7) for all traits and centelloside content was IPA hormone. OPDA showed a positive effect for branching and centelloside content, and *t*-Z only showed a positive correlation for centelloside content. IAA, on the other hand, had a strong negative effect (*r* < 0.7) on centelloside production, branching, and biomass production. ABA also had a negative effect on centelloside production and branching. IPA was strongly correlated (*r* > 0.8) with hairy root elongation and had a positive effect on centelloside content and biomass productivity at 28 days (Figure 5B; Supplementary Figure 8). OPDA and GA4 showed a negative correlation (*r* < 0.73) for all traits and centelloside content. ABA, SA, and *t*-Z were also negatively correlated except for root elongation. When the hormone content is compared between the two sampling times, the high amount of IPA hormone is repeatedly associated with a high content of centelloside, while the high amount of ABA is associated with low content.

### Prediction of production degree based on hormone profiles

Similarly, we investigated whether the hormonal profiles of transformed root lines could predict the degree of centelloside production. Four different classification machine learning algorithms were tested on a dataset (Supplementary Table 4) containing hormone content from the HIGH, MID, and LOW groups, and accuracy was used to track the models’ performance (Table 1). The most accurate models were RF and SVM, with 100% correct classification, and the least accurate model was LDA, with 82.57%. The sensitivity ranged from 50 to 100%, with SVM, RF, and ANN having the highest values and LDA having the lowest. The specificity range of the models was between 75 and 100%. The SVM model performed the best in classifying different degrees of centelloside production. The importance of each hormone for correct classification was calculated and ranked by the mean decrease in the Gini coefficient. The top 5 on this list were IPA, 2iP, ABA, IAA, and GA_4_ (Supplementary Figure 5B).

### Gene influence on hormone profiles

**Figure 6:**
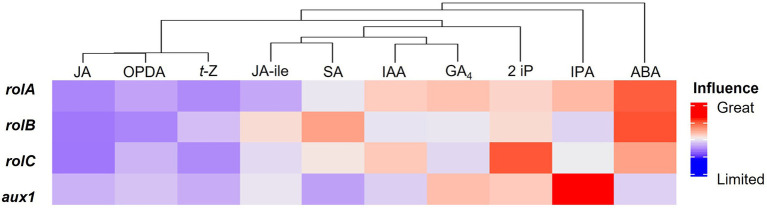
Global heatmap of the influence of *rol* and *aux1* genes on the hormone profile in hairy roots of *C. asiatica.*

We investigated how genes influence hormone profiles after studying how traits and centelloside content can be predicted by their gene expression or hormone profile. A multivariate multiple regression model was created for this purpose using the randomForestSRC R-package (see Materials and methods). The different hormones were treated as dependent variables in the model, while the genetic expression of *rol*A, *rol*B, *rol*C, and *aux*1 genes was treated as independent variables. The R-squared value for the model with all hormones was 0.788, which was used to measure the goodness of fit, the performance error of the model was 0.1998. The variable importance (VIMP) was used to compare the influence of genes on each hormone profile to aid in the interpretation of the multivariate regression model. Figure 6 shows a cluster analysis of hormones based on the influence of gene expression. ABA was most influenced by the behavior of *rol*B, followed by *rol*A and *rol*C, and *aux*1 had a low influence on its content. IPA was heavily influenced by *aux*1, followed by *rol*A. All genes had a greater than average influence on the 2 iP, with *rolC* having the greatest effect. The GA_4_ behavior was slightly affected by *rolA* and *aux*1. The genes *rolA* and *rolC* had a positive impact on IAA, while *aux1* and *rolB* had an average impact. Starting with SA, gene influence declined, followed by JA-ile, *t*-Z, OPDA, and JA as the hormone least influenced by gene expression. To improve the model’s fitness, the less influenced hormones by the *rol* or *aux* genes were eliminated one by one. When JA, OPDA, *t*-Z, JA-ile, SA, and GA_4_ were excluded, the best fitness was 0.837. In contrast, the absence of ABA resulted in the greatest decrease in model fitness, with an R-squared value of less than 0.3, followed by IPA and 2 iP. This abrupt decrease in R-squared value confirms the connection between the expression of these genes and the hormone profile of ABA, IPA, and 2 iP.

Y-randomization was implemented to prove accurate prediction potential of the model. We selected the top two most influenced hormones ABA, IPA and JA which was the lowest influenced by the gene expression to do this test. Individual models were built for each of these three hormones, the R-squared value was calculated and compared against the population of R-squared values obtained after 1,000 permutation (Supplementary Figure 10). As a result of this test, ABA and IPA showed to be accurate predicted by the regression model. Additionally, JA showed the overlapping of the simulated values with the original value of R-squared. The above results matched with the multivariate multiple regression model previously developed.

### Feeding experiments

**Figure 7:**
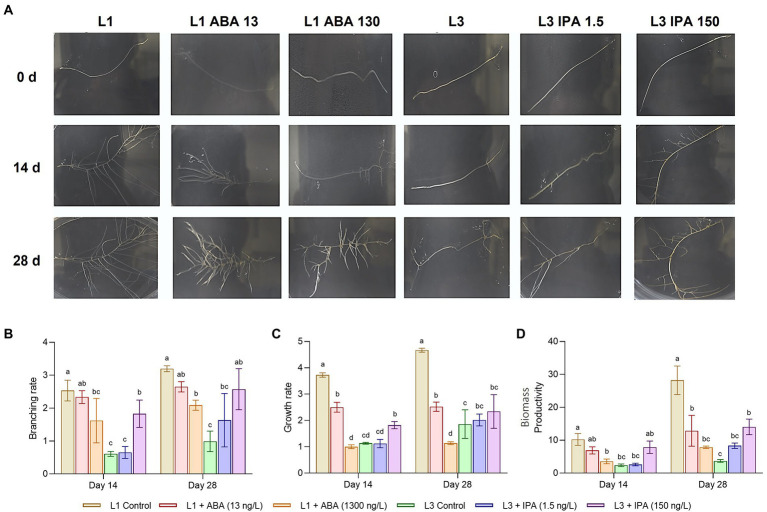
Feeding experiments on hairy roots lines, where L1 represent the HIGH group, while L3 the LOW group. **(A)** Developmental stages at different hormone concentration. **(B)** Branching rate at 14 and 28 days. **(C)** Growth rate. **(D)** Productivity of biomass. Data represent the mean ± SD of three replicates.

Figure 7A shows the development of root lines under the exogenous hormonal influence of ABA or IPA. The controls behaved consistently with the previous experiments, whereas the effects of ABA were perceived after 14 days at concentrations of 13 ng/l, causing significant decreases in the measured traits (Figures 7B–D) on the HIGH line. After 28 days, the lower concentration reduced branching, growth rate, and biomass productivity by 0.83, 0.54, and 0.45 times, respectively compared to the control. At 1,300 ng/l, the same traits were reduced 0.65, 0.24, and 0.28 times, respectively. When IPA was given to the LOW line, the opposite effect was seen; the effects on traits were visible at 28 days and concentrations of 150 ng/l. At the highest concentration, branching, growth rate, and biomass productivity were increased 2.60, 1.25, and 3.8 times after 28 days, respectively. Branching rate and biomass productivity were the most affected by ABA and IPA. Regarding centelloside content (Supplementary Figure 11), after 28 days on ABA treatment the HIGH line showed a decrease in 21% compared to the control when ABA was at 13 ng/l, while 74% of control when ABA was at 1300 ng/l. In contrast, the LOW line after 28 days on IPA treatment showed an increase in 1.76 times compared to the control when IPA was at 1.5 ng/l, while 3.21 times when IPA was 150 ng/l.

## Discussion

The hairy root syndrome is a disease that affects many plants and is caused by the infection and subsequent insertion of a fragment of the *A. rhizogenes* plasmid known as T-DNA (“transfer” DNA; Mauro et al., 2017). The *rol* genes, which are found in the TR-DNA region, are primarily responsible for the morphology and formation of these hairy roots (Ozyigit et al., 2013).

This type of *in vitro* culture system is useful for secondary metabolites biosynthesis and biotechnological production (Bonfill et al., 2015) since these cultures grow much faster than other types of *in vitro* cultures (Kim et al., 2007) and produce a spectrum of secondary metabolites similar to plant roots (Ruslan et al., 2012).

We observed the presence and expression of all *rol* and *aux* genes in all hairy lines studied (Supplementary Figure 3), which is similar to the work of Komarovská et al. (2009) in *Hypericum tomentosum*. and *Hypericum tetrapterum*, but differs from what was observed by Alpizar et al. (2008) in *Coffea arabica,* where the presence of any *aux* gene was found in all transformed lines. We selected the A. rhizogenes TL-DNA genes rolA, B and C because they have been shown to play the most relevant role in hairy root development (Sarkar et al., 2018; Bahramnejad et al., 2019), and the aux1 gene as a representative of the proper integration of the TR-DNA region in the transformed root lines (Mano and Nemoto, 2012). In our study, the expression level of all *rol* genes was higher than that of the *aux* gene (Supplementary Figure 3), which may be since only the presence of TL-DNA genes is required for long-term hairy root growth (Dessaux and Petit, 1994; Chriqui et al., 1996). The *rol*D was not analyzed because it was not detected in all *A. rhizogenes* strains (Pavlova et al., 2014).

When we compared all the hairy root lines or rhizoclones, we observed differences in growth and morphology that could be grouped into different categories, and it was thanks to the cultivation of these roots in a solid medium that the quantification of their traits was easily reproducible and simple to do. These differences between rhizoclones were attributed to variations in the nature, size, and number of T-DNA integrations into the host genome (Alpizar et al., 2008; Roychowdhury et al., 2015; Thwe et al., 2016). An evaluation with new methods to determine the number of copies of T-DNA integrated in the root lines could be an extension of the present study (Głowacka et al., 2016), which would allow identifying if the number of integrated copies is more relevant than the place of integration of the genes *rol* or *aux1*.

HIGH group lines had the highest expression value of the *rol*A gene, as well as the highest rooting rate, growth rate, biomass production, and centelloside content (Supplementary Figure 3). This gene is found in all Ri-plasmids and may be involved in the interaction with nucleic acids, which may be related to the regulation of gene expression in plants (Pavlova et al., 2014). The importance of this gene in secondary metabolism production was observed in *Rubia cordifolia* callus for anthraquinone production (Shkryl et al., 2008).

The evaluation of the gene expression using a multivariable analysis such as PCA allowed us to visualize in a simplified way the correlation and behavior of each of the characteristics of the different root lines (centellosides content, branching, elongation, and biomass production) over time. This analysis identified the grouping of root lines based on the degree of specific and recurrent gene expression. In general, low expression of *rol* and *aux*1 genes was associated with poor traits and low centelloside production. The genes *aux*1 and *rol*A were found to be more closely linked to lines with higher centelloside and trait content values. The *aux*1 gene is responsible for differences in hairy root growth and morphology (Ozyigit et al., 2013). This could be related to the line’s high rooting and growth rate in long-term culture (Figures 2, 4A), which was also observed by Lütken et al., 2017.

MID group lines had the highest levels of *rol*B and *rol*C gene expression (Figure 4A). The *rol*B is the most powerful inducer of secondary metabolism and the greatest suppressor of cell growth (Bulgakov, 2008). The MID group lines produced lower amount of centellosides than the HIGH group lines, which could be attributed to the high expression of *rol*C since it has previously been shown to have antagonistic effects between these two genes (Bulgakov et al., 2003). The *rol*C gene can stimulate the production of tropane alkaloids (Bonhomme et al., 2000), pyridine alkaloids (Palazon et al., 1998), ginsenosides (Bulgakov et al., 1998), and flavonoids (Ismail et al., 2017) in different *in vitro* culture systems, which differs somewhat from our studies.

The multivariable analysis (PCA) also exposed the behavior of the different hormones between traits and centelloside production, revealing a positive correlation with the IPA hormone content and a negative correlation with the ABA hormone. It is well known that ABA regulates numerous aspects of plant growth. Dicot plants deficient in this hormone have reduced seed dormancy and wilty phenotypes (Harris, 2015; Nambara, 2016). However, high levels of ABA have been shown to inhibit cell division in apical meristems and root elongation (Bai et al., 2009; Takatsuka and Umeda, 2014; Yang et al., 2014; Sun et al., 2018). This last scenario, in which high content is negatively correlated with elongation and branching, is consistent with our results, as is the low production of triterpene saponins. In terms of IPA, its high presence in hairy root lines was consistent with improved biomass production, elongation, and branching, and is supported by the abundance of lateral root meristems, which are one of the main sites of cytokinin synthesis (Nordström et al., 2004).

We focus on centelloside production for machine learning modeling since it is one of the most important parameters for biotechnological applications. The correlation analysis allowed us to consider the positive relationship between centelloside production and biomass production, elongation, and branching rate, allowing us to omit them from the models and simplify their execution. By discretizing the content of centellosides, it was possible to apply different supervised machine learning models, such as dimensionality reduction (LDA; Zhao et al., 2020), instance-based (SVM; Cristianini and Ricci, 2008), ensemble methods (RF; Liaw and Wiener, 2002), and artificial neural network (ANN; Venables and Ripley, 2002). The reason for selecting these models was due to their nature, as mentioned above. In general, RF and SVM performed the best with the data presented in this work, correctly classifying the samples into the classes proposed. The LDA, on the other hand, produced the worst results, which could be attributed to the fact that the data distribution was not normal for all variables. The data distribution was identified by Shapiro–Wilk test and the results are shown in Supplementary Table 5.

Multiple multivariate regression models were used to understand how certain hormones and gene expression levels (*rol* and *aux1* genes) interact to coordinate root growth and development. This allowed us to simultaneously evaluate the influence of each level of genetic expression on the profile of each hormone studied. The random forest method has the advantage of being able to work with data whose distribution may or may not be normal; it evaluated the importance of each variable within the model, allowing identifying the degree of influence of genes on each of the growth regulators. Feeding experiments validated the model by demonstrating that the analyzed phytohormones (IPA and ABA) were determinants in increasing the high producer line and decreasing the low producer line, as well as influencing centelloside production.

The use of this biotechnological platform together with machine learning techniques resulted in the implementation of models that allow us to discriminate root lines based on their level of production of secondary metabolites such as centelloside, with random forest outperforming all others. This discrimination was made possible by using gene expression levels of the *rol* and *aux1* genes, as well as hormone profiles. Furthermore, the degree of influence of each gene on the individual profile of each hormone studied was determined, with IPA and ABA being the most affected due to the action of the *rol* and *aux1* genes. Finally, the results of the gene influence analysis on these two specific hormones were successfully tested with feeding experiments aimed at reversing the effect on root morphology and centelloside content.

## Data availability statement

The original contributions presented in the study are included in the article/Supplementary material, further inquiries can be directed to the corresponding authors.

## Author contributions

MB, JP, and DH-M designed the research. MA, MM, and SM-B determined the hormone profile. MA, ML, and PG build neural networks models. MA and DH-M build the others machine learning models. MA performed tissue culture and metabolites determination. All authors contributed to the article and approved the submitted version.

## Funding

This work was partially funded by the Spanish Ministry of Science and Innovation, with project number PID2020-113438RB-I00, and by the Catalan Government, with project number 2017SGR980.

## Conflict of interest

The authors declare that the research was conducted in the absence of any commercial or financial relationships that could be construed as a potential conflict of interest.

## Publisher’s note

All claims expressed in this article are solely those of the authors and do not necessarily represent those of their affiliated organizations, or those of the publisher, the editors and the reviewers. Any product that may be evaluated in this article, or claim that may be made by its manufacturer, is not guaranteed or endorsed by the publisher.

## Footnotes

^1^https://www.bioinformatics.nl/cgi-bin/primer3plus/primer3plus.cgi (Accessed August 22, 2022).

^2^www.graphpad.com (Accessed August 22, 2022).
