# Supplementary material for: Using machine learning to link the influence of transferred Agrobacterium rhizogenes genes to the hormone profile and morphological traits in Centella asiatica hairy roots
Source: Front Plant Sci. 2022 Sep 2;13:1001023. doi: 10.3389/fpls.2022.1001023 (PMC9479193; doi:10.3389/fpls.2022.1001023)
Supplement: Supplementary file 1 [file Data_Sheet_1.PDF]

Supplementary material

Using machine learning to link the influence of transferred *Agrobacterium rhizogenes* genes to the hormone profile and morphological traits in *Centella asiatica* hairy roots.

Miguel Angel Alcalde[1], Maren Müller[2], Sergi Munné-Bosch[2], Mariana Landín[3], Pedro Pablo Gallego[4], Mercedes Bonfill[1], Javier Palazon[1], Diego Hidalgo-Martinez[1,5]

[1]Department of Biology, Healthcare and the Environment. Faculty of Pharmacy and Food Sciences. University of Barcelona. Spain

[2]Department of Evolutionary Biology, Ecology and Environmental Sciences, Faculty of Biology. University of Barcelona. Spain

[3]Pharmacology, Pharmacy and Pharmaceutical Technology Department, Grupo I+D Farma (GI-1645), Faculty of Pharmacy, University of Santiago, E-15782 Santiago de Compostela, Spain

[4]Agrobiotech for Health, Plant Biology and Soil Science Department, Faculty of Biology, University of Vigo, E-36310 Vigo, Spain

[5]Department of Plant and Microbial Biology, University of California, Berkeley, California 94720-3102, United States.

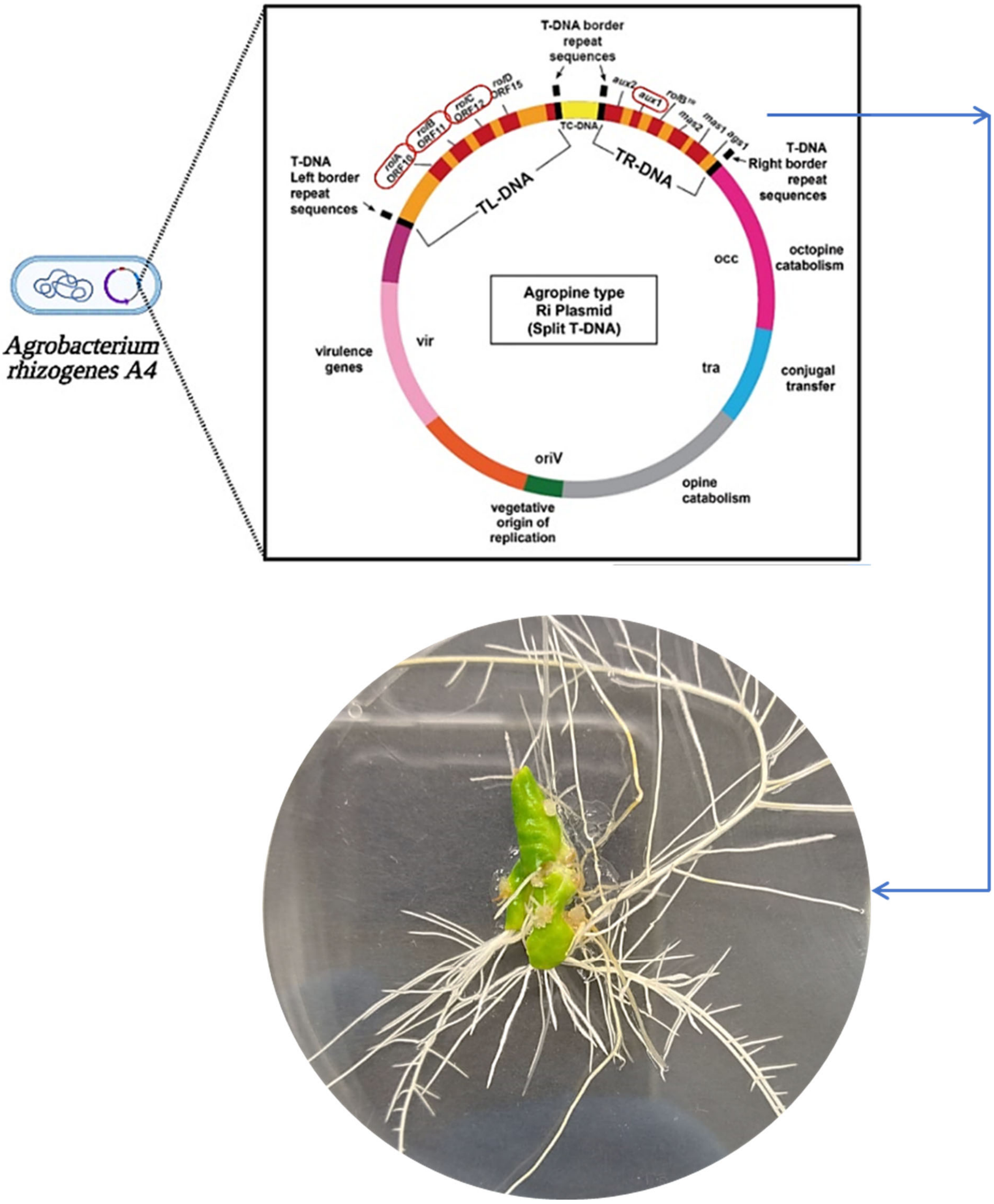

Figure 1. Ri plasmid from *Agrobacterium rhizogenes* A4 and photograph of hairy root induction from infected explants. Red circles are remarking genes in study. The Image of plasmid was modified from Ozyigit *et al.* (2013).

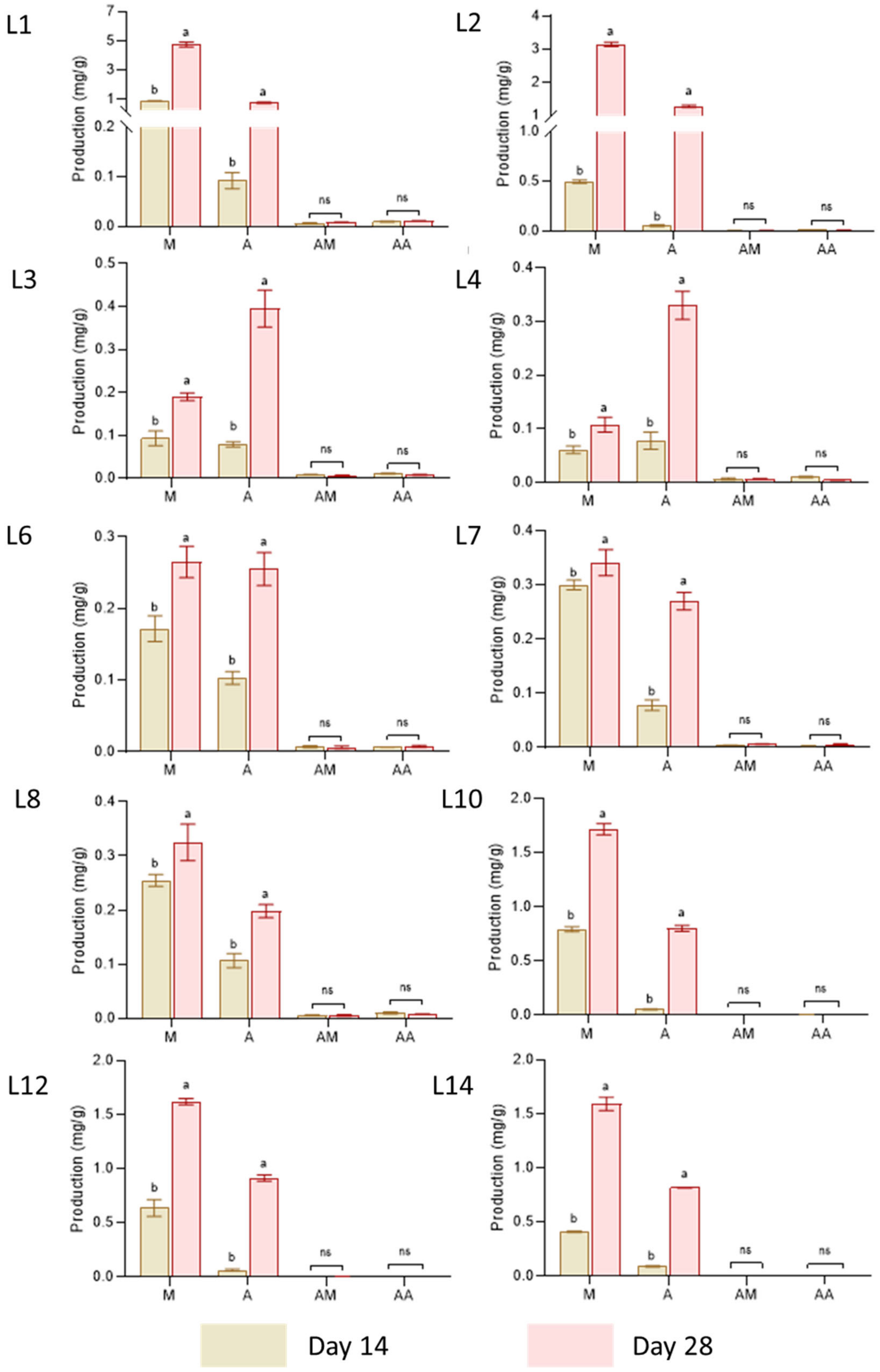

Figure 2. Specific centelloside production (mg/g DW). Madecassoside (M), Asiaticoside (A), Madecassic acid (AM) and Asiatic acid (AA) of *C. asiatica* hairy root lines at day 14 and 28.

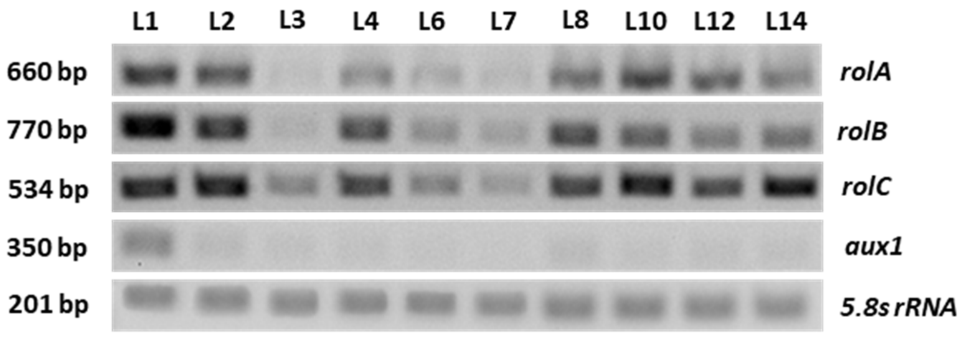

Figure 3. Semiquantitative reverse transcriptase PCR analysis. Expression of rol (rolA, rolB and rolC) and aux (aux1) genes in different *C. asiatica* hairy root lines in MS basal medium after 28 days of growth. 5.8s rRNA was used as housekeeping gene.

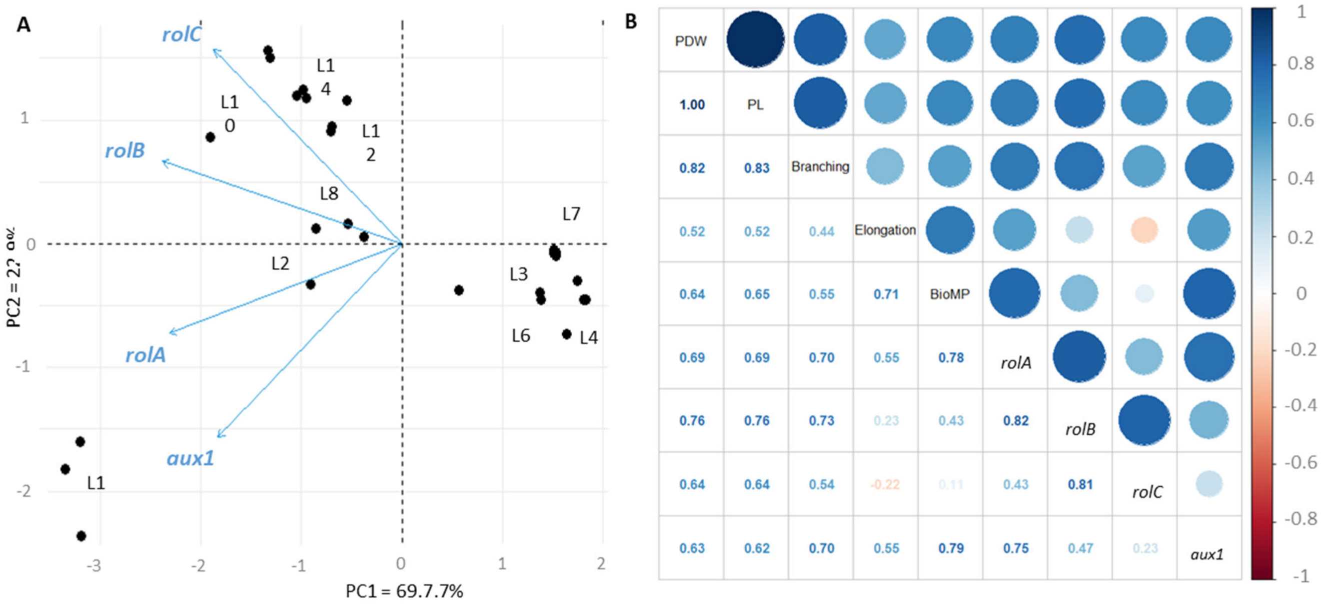

Figure 4. Gene expression analysis and correlation study with morphological parameters and centelloside production of *C. asiatica* hairy roots at day 14. (A) Principal Component Analysis of genes studied. (B) Correlation study of gene expression, morphological parameters and centelloside production. Elongation, refers to growth rate, while BioMP refers to productivity of biomass

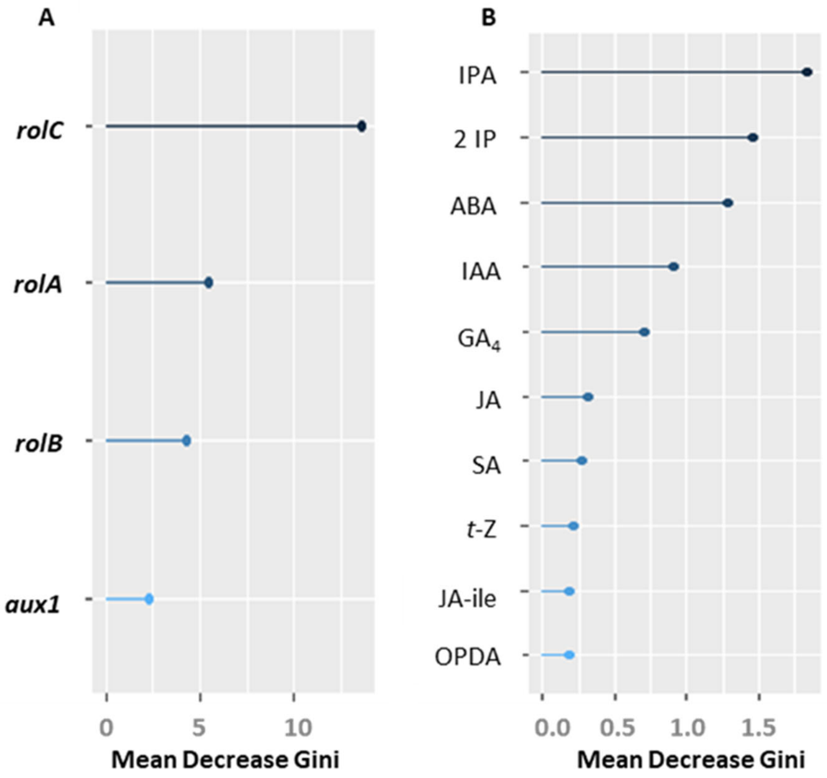

Figure 5. Effect of gene expression and hormone profiling in hairy root lines. (A) Importance of genes studied in hairy root lines. (B) Importance of plant hormones analyzed in hairy root lines.

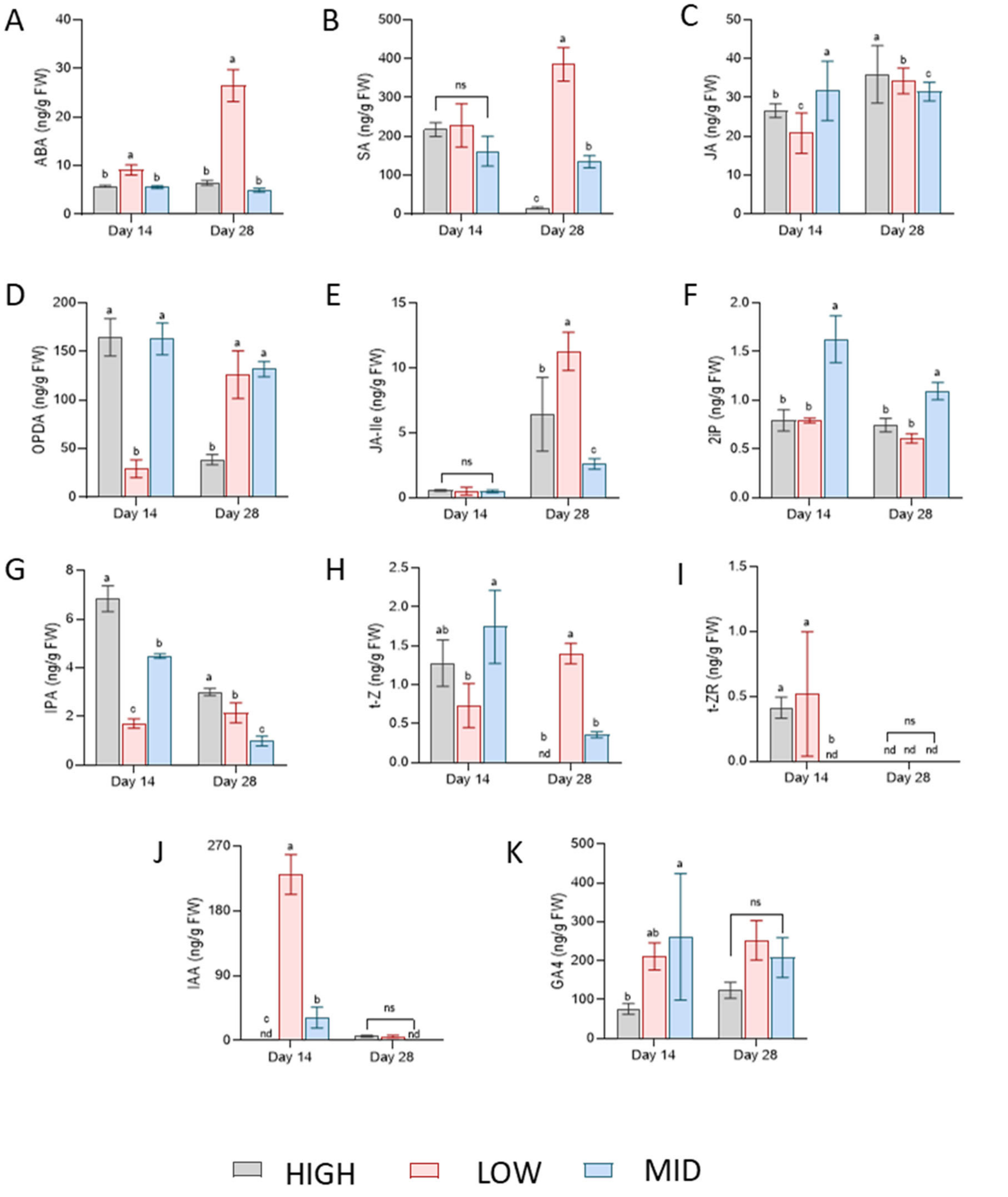

Figure 6. Phytohormone concentration (ng/g FW) in *C. asiatica* hairy root lines. (A) ABA, (B) SA, (C) JA, (D) OPDA, (E) JA-Ile, (F) 2iP, (G) IPA, (H) t-Z, (I) *t*-ZR, (J) IAA, and (K) GA_4. Data represent the mean ± SD of three replicates. Different letters show significant differences between hairy root lines at each time point. nd=no determinated. ns=no significance.

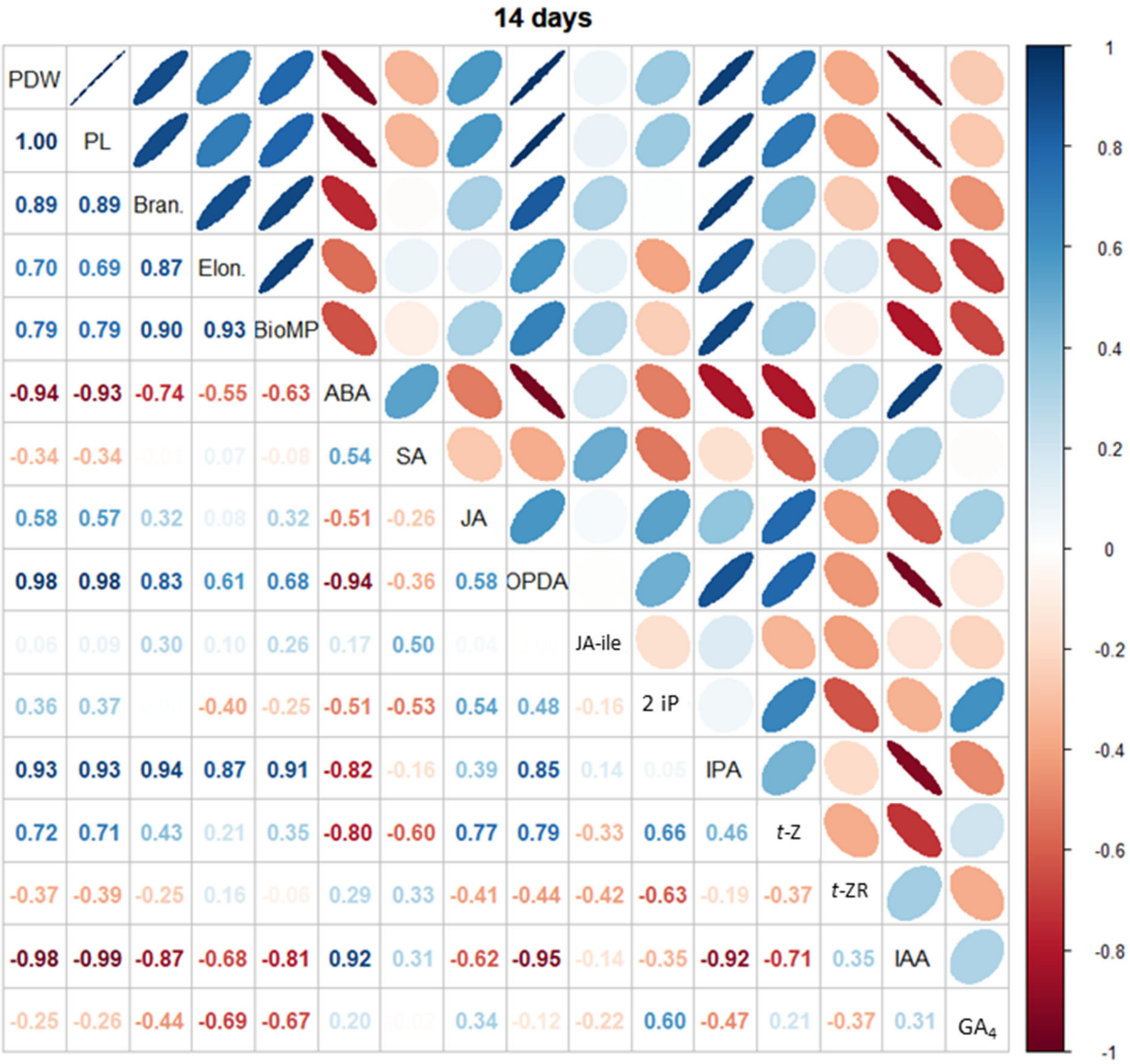

Figure 7. Correlation study of morphological parameters, production rates and plant hormone values at 14-day culture. Elon., refers to growth rate, while BioMP refers to biomass productivity.

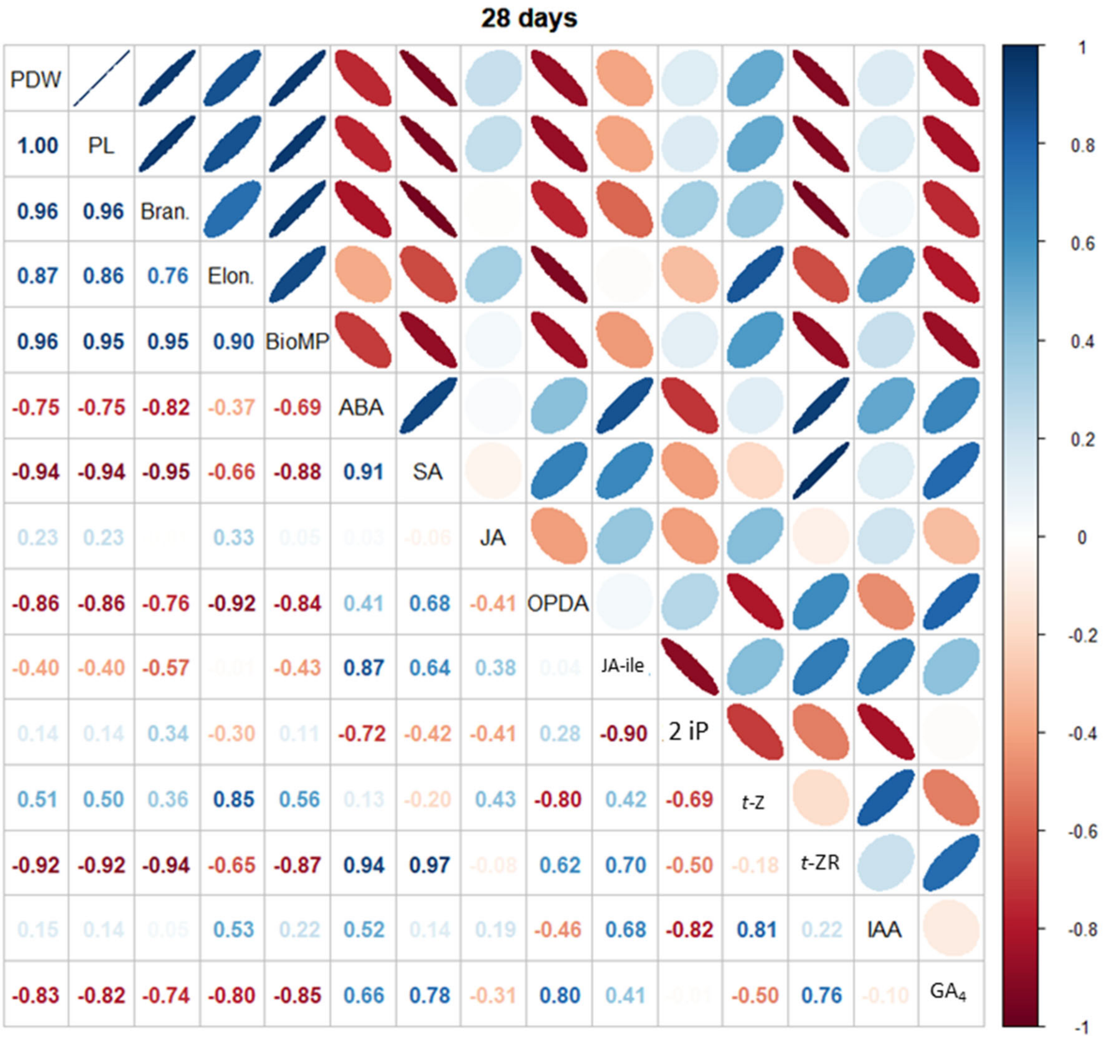

Figure 8. Correlation study of morphological parameters, production rates and plant hormone values at 28-day culture. Elon., refers to growth rate, while BioMP refers to biomass productivity.

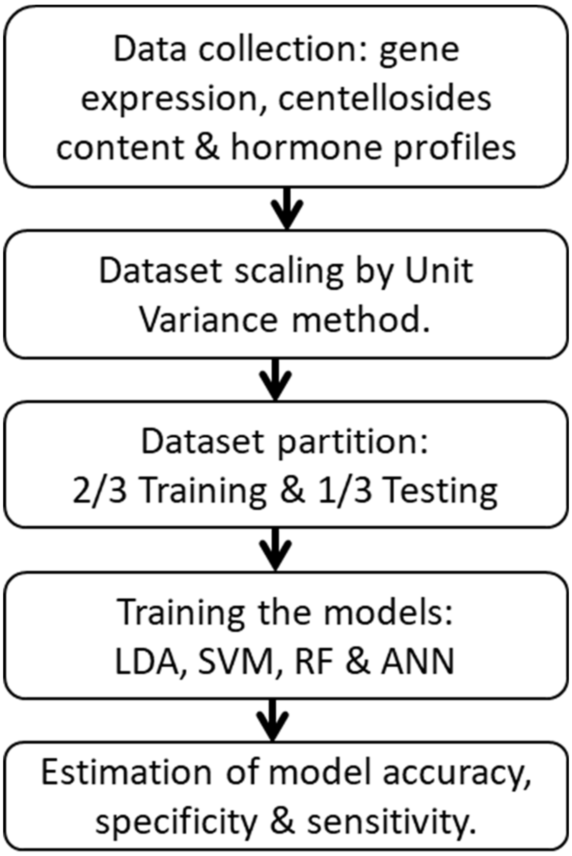

Figure 9. Machine Learning model workflow.

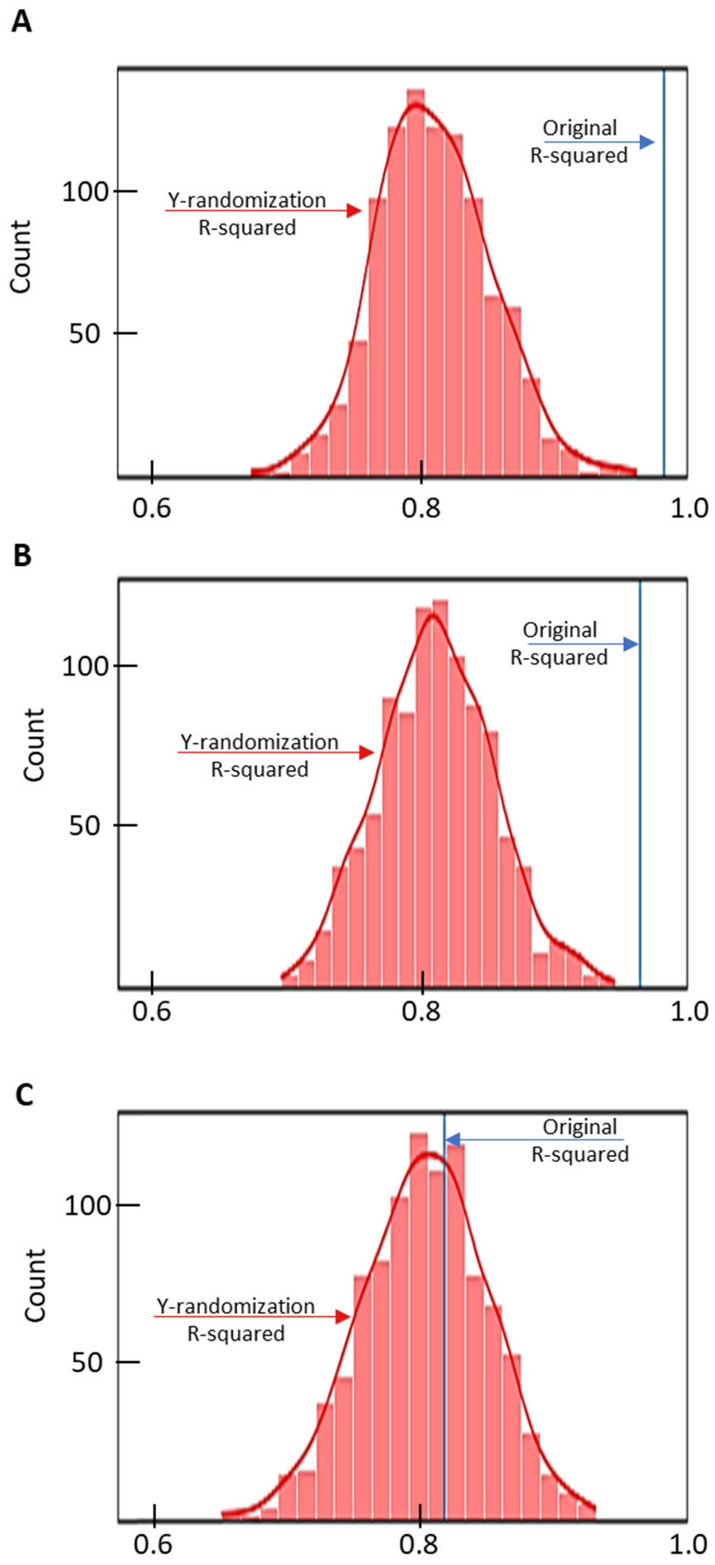

Figure 10. Y-randomization analysis for (A) ABA. (B) IPA. (C) JA. Blue line indicate the R-squared value with the origianal data. Red line and bars indicate the distribution of R-squered values after 1000 permutation.

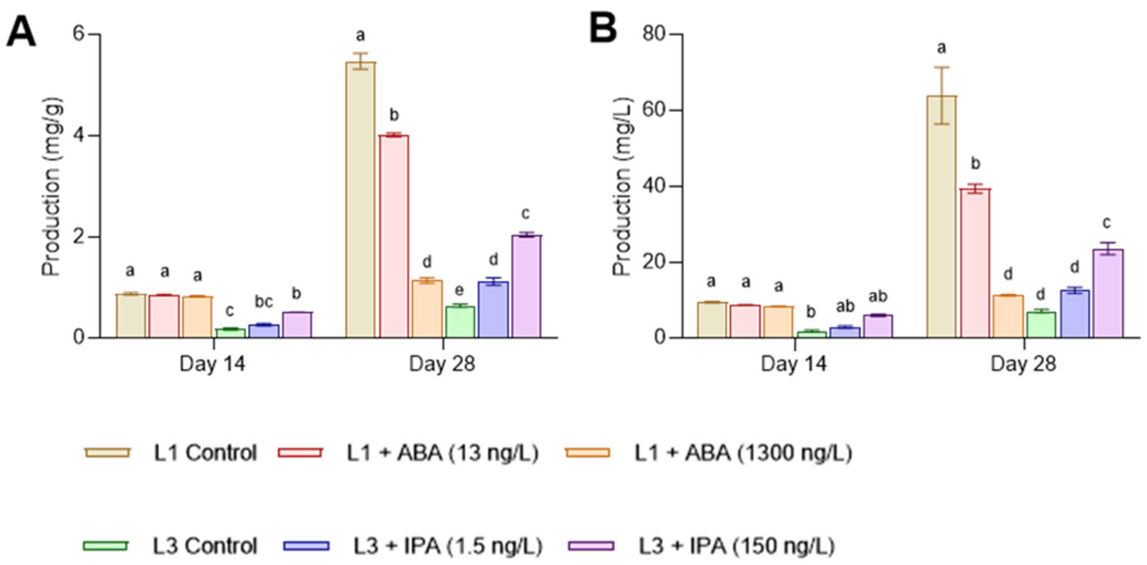

Figure 11. Centelloside content in feeding experiments on hairy roots lines, where L1 represent the HIGH group, while L3 the LOW group. (A) Production (mg/g DW) and (B) Production (mg/L of media) at 14 and 28 day. Data represent the mean ± SD of three replicates. Different letters show significant differences (α=0.05). between treatments.

Table 1. Primers used for confirmation of transformed roots

Gene		Primer sequence	Temperature melting (°C)	Amplicon size (pb)	Reference – Accession number
aux1	Forward	5´-TTCGAAGGAAGCTTGTCAGAA-3´	60	350	KX986281.1
	Reverse	5´-CTTAAATCCGTGTGACCATAG-3´			(*A. rhizogenes* A4)
rolA	Forward	5´-TGGAATTAGCCGGACTAAAC-3´	60	660	KX986281.1
	Reverse	5´-GCGTACGTTGTAATGTGTTG-3´			(*A. rhizogenes* A4)
rolB	Forward	5´-AGTTCAAGTCGGCTTTAGGC-3´	60	770	KX986281.1
	Reverse	5´-TCCACGATTTCAACCAGTAG-3´			(*A. rhizogenes* A4)
rolC	Forward	5´-TAACATGGCTGAAGACGACC-3´	60	534	KX986281.1
	Reverse	5´-AAACTTGCACTCGCCATGCC-3´			(*A. rhizogenes* A4)
5.8s rRNA	Forward	5´-CGGCAACGGATATCTCGGCTCT-3´	66	201	OK440405.1 – Mangas et al.,
	Reverse	5´-TCCGCCCCGACCCCTTTC-3´			2008 [7] (*Arabidopsis thaliana*)

Table 2. Gradient used for HPLC separation of the centellosides

Time (min)	Flow (mL/min)	Aqueous solvent (%)	Organic solvent (%)
**0**	1	80	20
**15**	1	62	38
**30**	1	30	70
**35**	1	30	70
**37**	1	80	20
**45**	1	80	20

Table 3. Dataset for prediction of production degree based on gene expression.

Class	Line	Day	PDW	PL	Branching	Growth rate	BioMP	RolA	RolB	RolC	Aux1
HIGH	L1	14	0.983	12	2.1	3.6	8.6	12373	10609	7093	9827
HIGH	L1	14	0.975	12.2	2.5	4.3	10	11208	10441	7639	9727
HIGH	L1	14	0.926	11.6	2.7	3.8	10.7	12374	8242	7099	11404
HIGH	L2	14	0.544	6.9	1.5	4.2	4.3	8618	9347	5112	288
HIGH	L2	14	0.564	7.1	1.3	3.5	5.7	1471	1938	2379	368
HIGH	L2	14	0.55	7	1.6	3.6	4.3	1472	1932	2370	368
LOW	L3	14	0.173	1.8	0.7	1.4	2.1	633	1930	4736	1156
LOW	L3	14	0.191	2.1	0.9	1.6	2.1	603	1939	4835	1107
LOW	L3	14	0.153	1.9	1.1	1.1	2.9	630	1923	4631	1138
LOW	L4	14	0.138	1.7	1.1	1.1	1.4	632	1837	4777	1096
LOW	L4	14	0.163	1.7	1.3	1.3	1.4	1472	1936	2379	368
LOW	L4	14	0.118	1.3	1.4	1.3	3.6	2354	2769	3184	818
LOW	L6	14	0.286	3.6	1.1	1.4	5	2748	2350	3199	792
LOW	L6	14	0.276	3.4	0.9	1.9	4.3	1071	1556	3506	732
LOW	L6	14	0.264	3.4	1.1	1.6	5	7081	7248	5441	678
LOW	L7	14	0.372	4.7	1.4	2.2	5	7603	8104	5375	294
LOW	L7	14	0.382	4.7	1.4	2.5	3.6	1518	1998	2391	377
LOW	L7	14	0.378	4.8	1.2	2.3	4.3	1472	1931	2379	368
HIGH	L8	14	0.36	4.5	1.8	2.4	3.6	7359	8259	5522	2733
HIGH	L8	14	0.358	4.3	1.2	2.3	2.9	1472	1933	2379	1638
HIGH	L8	14	0.368	4.6	1.5	2.1	3.6	3981	5077	3763	1463
MID	L10	14	0.853	10.6	1.8	1.6	2.9	4446	9504	11512	1520
MID	L10	14	0.822	10.5	1.6	2	7.1	8999	9059	10834	1940
MID	L10	14	0.857	10.5	1.6	1.7	5	4447	9506	11712	1441
MID	L12	14	0.78	9.8	2.2	1.8	2.1	3962	8266	9050	1515
MID	L12	14	0.681	8.6	2.2	1.3	2.9	3979	7690	9460	1857
MID	L12	14	0.639	8	1.9	1.8	3.6	3149	8242	9351	1156
MID	L14	14	0.5	6.4	1.7	1.1	2.9	4826	8245	10244	1257
MID	L14	14	0.486	6	1.7	1.3	2.1	4824	8243	10535	1196
MID	L14	14	0.511	6.4	1.6	1	3.6	4837	8291	10630	1520
HIGH	L1	28	5.25	64.7	3.4	4.7	41.4	12219	8441	7123	11404
HIGH	L1	28	5.64	70.8	3.1	4.9	37.5	10296	11686	7795	8656
HIGH	L1	28	5.57	70.7	2.9	4.4	28.6	12374	10831	7459	9720
HIGH	L2	28	4.42	54.2	2.1	4.7	23.6	9362	7378	5210	2224
HIGH	L2	28	4.37	54.4	2.2	4.3	25.4	8324	11175	4877	2611
HIGH	L2	28	4.48	57.4	2.5	3.9	18.2	8950	9034	5044	2289
LOW	L3	28	0.56	7	1	2.9	6.8	667	1169	4766	1267
LOW	L3	28	0.63	8.1	1	2.3	4.3	777	1144	4443	1641
LOW	L3	28	0.56	7	1.2	2.2	2.9	633	1634	4605	1472
LOW	L4	28	0.43	5.2	1.5	2.6	2.1	7633	5527	4471	1616
LOW	L4	28	0.47	5.5	1.6	2.6	1.1	5943	6171	4587	1775
LOW	L4	28	0.42	5.1	1.7	2.7	2.5	7038	5643	4529	1715
LOW	L6	28	0.54	6.9	1.6	2.5	6.4	2777	2375	3576	914
LOW	L6	28	0.54	6.7	2	2.2	8.9	3506	3262	3355	836
LOW	L6	28	0.48	6	2.2	2.1	7.1	2816	3349	3466	901
LOW	L7	28	0.65	8	1.5	2.9	7.1	1738	1769	2379	454
LOW	L7	28	0.6	7.3	1.7	2.8	6.4	2219	1718	2691	368
LOW	L7	28	0.58	7.3	1.6	2.5	3.9	1713	1594	2535	663
HIGH	L8	28	0.53	6.8	1.7	3	10.7	7506	7346	5663	2998
HIGH	L8	28	0.54	6.8	2.2	2.8	14.3	8098	8693	5458	2634
HIGH	L8	28	0.5	6.4	2.2	3.1	12.1	7293	7985	5561	2754
MID	L10	28	2.51	32.4	2.4	2.1	15.7	5980	10560	13379	1374
MID	L10	28	2.55	31.9	2	2.6	18.2	5550	12186	12665	1314
MID	L10	28	2.49	31.3	2.1	2.2	12.5	5710	11962	13022	1206
MID	L12	28	2.52	31.6	2.9	1.9	8.2	3916	7582	9312	1899
MID	L12	28	2.5	31.7	2.6	1.7	3.9	4560	8720	9483	1495
MID	L12	28	2.57	32.7	2.6	2.4	6.1	3543	8242	9397	1501
MID	L14	28	2.44	30.6	2	1.1	5.4	5117	6837	11080	2037
MID	L14	28	2.45	30.7	2	1.6	3.9	5211	8091	10573	1546
MID	L14	28	2.34	30	1.8	1.5	3.2	4724	7942	10827	1811

Table 4. Dataset for prediction of production degree based on hormone profiles.

Class	Day	ABA	SA	JA	OPDA	Ile-JA	2-iP	IPA	*t*-Z	*t*-ZR	IAA	GA4
HIGH	14	5.88	215.04	27.62	157.46	0.53	0.92	7.30	1.04	0.47	0.00	81.90
HIGH	14	5.91	236.97	27.76	186.40	0.52	0.70	6.27	1.61	0.45	0.00	86.58
HIGH	14	5.59	201.18	24.58	150.12	0.63	0.76	6.97	1.19	0.32	0.00	61.04
LOW	14	8.16	211.28	22.26	28.22	0.28	0.82	1.56	1.04	1.07	213.60	173.86
LOW	14	9.00	183.05	15.11	38.95	0.38	0.78	1.93	0.68	0.29	262.23	217.65
LOW	14	10.25	290.80	25.19	20.56	0.84	0.78	1.66	0.47	0.19	215.43	242.90
MID	14	5.26	197.92	25.11	178.33	0.52	1.86	4.38	1.50	0.00	43.46	290.38
MID	14	5.67	121.71	30.03	145.89	0.57	1.38	4.54	1.67	0.00	15.67	86.40
MID	14	5.90	165.78	40.04	164.91	0.38	1.65	4.54	2.06	0.00	37.05	407.67
HIGH	28	6.20	18.50	29.95	42.65	3.16	0.82	3.07	0.00	0.00	7.23	101.40
HIGH	28	7.06	14.34	33.72	40.42	7.83	0.69	3.11	0.00	0.00	5.06	140.81
HIGH	28	6.17	15.65	44.21	32.81	8.32	0.72	2.83	0.00	0.00	5.30	130.00
LOW	28	25.77	434.46	37.21	126.34	10.74	0.65	2.60	1.25	0.00	4.92	238.72
LOW	28	23.66	354.31	34.90	101.45	10.17	0.56	2.04	1.49	0.00	4.34	210.99
LOW	28	30.09	367.40	30.69	150.44	12.96	0.62	1.82	1.46	0.00	7.39	309.17
MID	28	5.24	152.46	28.88	122.97	2.16	1.19	0.94	0.40	0.00	0.00	262.27
MID	28	5.13	129.30	32.19	137.81	2.96	1.02	0.90	0.34	0.00	0.00	161.13
MID	28	4.48	122.92	33.49	135.20	2.70	1.08	1.16	0.34	0.00	0.00	200.94

Table 5. Data distribution.

Variable	14 days		28 days	
	Shapiro-Wilk (W)	p(normal)	Shapiro-Wilk (W)	p(normal)
**ABA**	0.7885	1.51E-02	0.7304	3.21E-03
**SA**	0.9694	8.90E-01	0.8535	8.15E-02
**JA**	0.9228	4.16E-01	0.8855	1.79E-01
**OPDA**	0.7884	1.51E-02	0.8308	4.56E-02
**Ile-JA**	0.9443	6.28E-01	0.8806	1.59E-01
**2-iP**	0.7896	1.55E-02	0.8997	2.50E-01
**IPA**	0.8883	1.92E-01	0.8873	1.87E-01
***t*-Z**	0.9751	9.34E-01	0.802	2.15E-02
***t*-ZR**	0.8436	6.33E-02	0.3898	3.22E-07
**IAA**	0.7595	6.99E-03	0.8323	4.73E-02
**GA4**	0.8979	2.40E-01	0.9734	9.22E-01
**PDW**	0.931	5.21E-02	0.8026	7.32E-05
**PL**	0.9324	5.69E-02	0.8065	8.63E-05
**Branching**	0.9653	4.19E-01	0.9743	6.61E-01
**Growth rate**	0.8583	9.31E-04	0.9081	1.34E-02
**BioMP**	0.8507	6.41E-04	0.8218	1.68E-04
**RolA**	0.8746	2.12E-03	0.9668	4.57E-01
**RolB**	0.7973	5.85E-05	0.9201	2.70E-02
**RolC**	0.8921	5.41E-03	0.8998	8.32E-03
**Aux1**	0.537	1.35E-08	0.5983	6.96E-08

References

Ozyigit, I. I., Dogan, I., and Artam Tarhan, E. (2013). Agrobacterium rhizogenes-Mediated Transformation and Its Biotechnological Applications in Crops. *Crop Improv. New Approaches Mod. Tech.*, 1–48. doi: 10.1007/978-1-4614-7028-1_1.
